# The potential role of three-dimensional surface imaging as a tool to evaluate aesthetic outcome after Breast Conserving Therapy (BCT)

**DOI:** 10.1007/s10549-017-4256-y

**Published:** 2017-04-26

**Authors:** Rachel L. O’Connell, Rosa Di Micco, Komel Khabra, Lisa Wolf, Nandita deSouza, Nicola Roche, Peter A. Barry, Anna M. Kirby, Jennifer E. Rusby

**Affiliations:** 1 Department of Breast Surgery, The Royal Marsden NHS Foundation Trust, Orchard House, Downs Road, Sutton, SM2 5PT UK; 2 Department of Medicine and Surgery, University of Naples Federico II, Via S. Pansini 5, 80131 Naples, Italy; 3 Department of Statistics, The Royal Marsden NHS Foundation Trust, Sutton, UK; 4 Cancer Research UK Cancer Imaging Centre, Institute of Cancer Research and Royal Marsden NHS Foundation Trust, Sutton, UK; 5 Department of Clinical Oncology, The Royal Marsden NHS Foundation Trust, Sutton, UK

**Keywords:** Breast cancer, Patient reported outcome measures, Aesthetic outcome

## Abstract

**Purpose:**

To establish whether objective measurements of symmetry of volume and shape using three-dimensional surface imaging (3D-SI) can be used as surrogate markers of aesthetic outcome in patients who have undergone breast conserving therapy (BCT).

**Methods:**

Women who had undergone unilateral BCT in the preceding 1–6 years were invited to participate. Participants completed a satisfaction questionnaire (BREAST-Q) and underwent 3D-SI. Volume and surface symmetry were measured on the images. Assessment of aesthetic outcome was undertaken by a panel of clinicians. The Kruskal–Wallis test was used to assess the relationship between volume and shape symmetry measurements with the panel score. Spearman’s rho correlations were used to assess the relationship between the measurements and patient satisfaction.

**Results:**

200 women participated. Median volume symmetry was 87% (IQR 78–93) and shape symmetry was 5.9 mm (IQR 4.2–8.0). The participants were grouped according to panel assessment of aesthetic outcome (poor, fair, good, excellent) and the median volume and shape symmetry was calculated for each group. Volume symmetry significantly differed between the groups. Post hoc pairwise comparisons demonstrated that these differences existed between panel scores of fair versus good and good versus excellent. Median shape symmetry also differed according to patient panel groups with four significant pairwise comparisons between poor versus good, poor versus excellent, fair versus good and fair versus excellent. There was a significant but weak correlation of both volume symmetry and surface asymmetry with BREAST-Q scores (correlation coefficients 0.187 and −0.229, respectively).

**Conclusion:**

Breast volume and shape symmetry are both associated with panel assessment scores and patient satisfaction. The objective volume and shape symmetry measures were strongly associated with panel assessment scores, such that a 3D-SI tool could replace panel assessment as a faster and more objective method of evaluating aesthetic outcomes.

## Introduction

In the UK, around 30,000 women per year undergo breast conserving treatment (BCT) for breast cancer [1]. The combination of surgery and radiotherapy has achieved good local control rates [2, 3] and is equivalent to mastectomy. However, the technical challenges of completely excising tumour whilst also re-shaping breast tissue to provide an aesthetically acceptable outcome yield variable cosmetic results and variable patient satisfaction. Therefore, although the long-term success of BCT is measured primarily by local control, increased survivorship (87% at 5 years) [4], demands that the physical and psychological effects of treatment, especially long-term effects, are addressed. Dissatisfaction with the appearance after treatment acts as a constant reminder of the disease and affects a woman’s psychological wellbeing [5–7].

Assessment of aesthetic outcome has, to date, remained a challenge for breast and plastic surgeons alike. There is no gold standard or consensus on which factors should be assessed and who should undertake the analysis [8]. The mainstays of assessment are subjective, using either patient-reported outcome measures (PROMs) [9–12] or panel assessment of appearance [13, 14]. More recently, objective assessments have been developed in the form of the Breast Analysing Tool (BAT^©^) [15] and the Breast Cancer Conservative Treatment cosmetic result software (BCCT.core) [16, 17] whereby a single photograph (anterior view) of the patient is analysed to give an aesthetic outcome score between 1 and 4.

Three-dimensional surface imaging (3D-SI) has been used as a marketing tool in aesthetic breast surgery. Three-dimensional (3D) images are taken and manipulated to illustrate to potential patients how they may appear after augmentation or mammoplasty/mastopexy. A survey of 1067 plastic surgeons in America revealed that 15% of surgeons are using 3D-SI technology in their practice [18]. We have reported on the use of 3D-SI as a research and clinical tool in aesthetic, oncoplastic and reconstructive breast surgery [19] using linear distances and 3D measurements such as volume and symmetry. Several other studies have validated its use in volume measurement [20–29] as a surgical planning tool. The aim of this study was to establish whether symmetry of volume and shape on 3D-SI following BCT can be used as surrogate markers of aesthetic outcome (as judged by a panel assessment or by PROMs).

## Methods

### Patient recruitment

Research Ethical Committee (REC) approval was obtained (ClinicalTrials.gov Identifier: NCT02304614). Women aged ≥18 years who had undergone BCT (wide local excision and adjuvant whole breast radiotherapy) for an invasive or in situ carcinoma in our unit (four permanent surgeons) between one and six years before the start of the study met the inclusion criteria. The following patients were excluded: those who developed recurrent (local or distant) disease since BCT, those who had previously or subsequently undergone surgery to the index or contralateral breast and those who were unable to undergo 3D-SI (e.g. unable to stand for 5 min).

Patients were invited to participate in the study by letter, followed up by a telephone call. Those who agreed to participate were offered an appointment for 3D-SI before or after their mammogram or at another mutually convenient time. The 3D-SI images were taken in the medical photography department.

### 3D-SI breast imaging

**Fig. 1:**
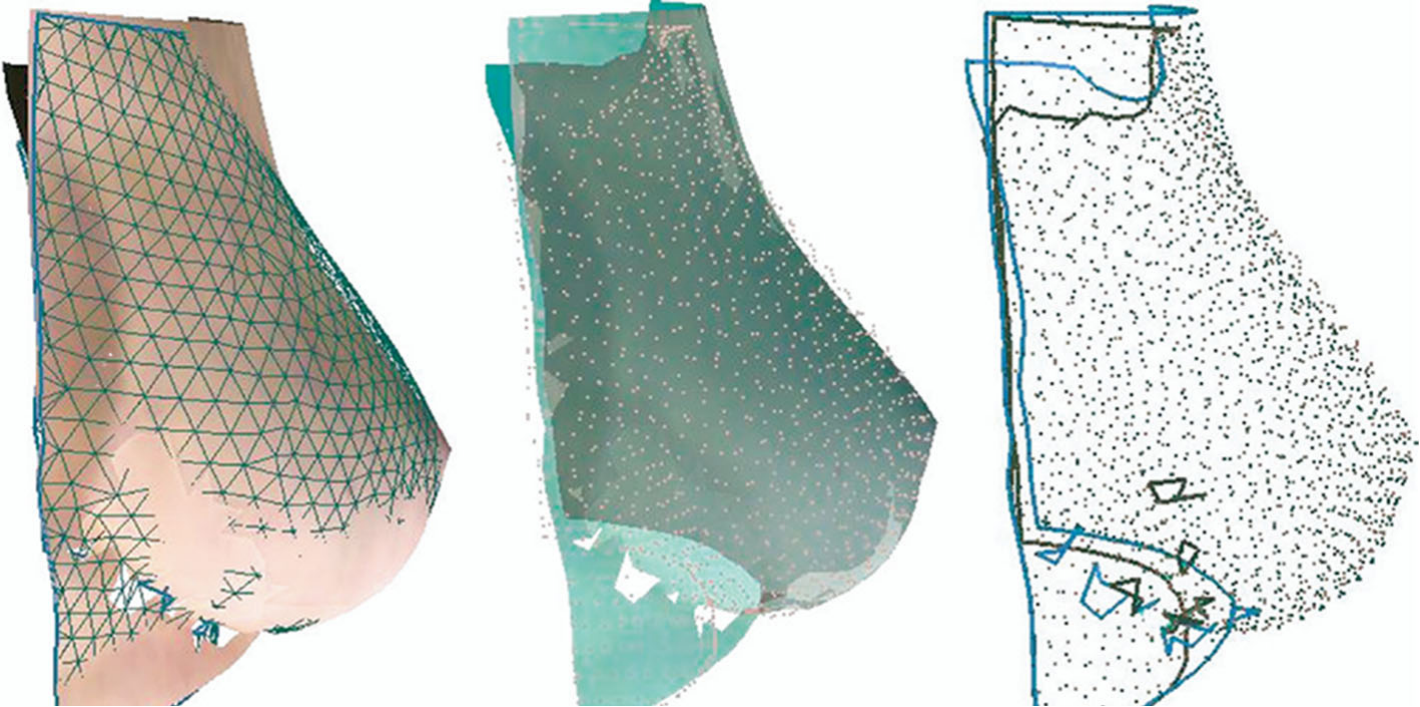
Example of calculation of shape symmetry using the root mean squared (RMS). Reproduced with the permission of Canfield Scientific. The *left* breast image is reflected onto the *right*. In the first image a geometric pattern is applied to one breast image. The perpendicular distances from all the interception points of the grid to the other breast is calculated (*second* and *third* image)

Participants were imaged using the Vectra-XT 3D-SI system with their hands on hips. Volume symmetry was calculated by dividing the volume of the smaller breast by the volume of the larger breast and converting to a percentage value. The closer the result to 100, the more symmetrical the breasts were in terms of volume. Shape symmetry was calculated by bisecting the 3D image vertically along the midline and reflecting the image of the left breast onto the right. The root mean squared (RMS) of the distances between the two superimposed breast surfaces was calculated in millimetres (Fig. 1). The lower the score the more symmetrical the breasts are in terms of shape.

### Panel assessment

The panel consisted of four members, two breast surgeons, one clinical oncologist and one breast care nurse. Panellists were asked to rate the participants’ images according to the 4-point Harvard cosmesis scale (Table 1). Initially, the scoring system was to assess the effect of radiotherapy [13] but has since then been adapted to a cosmetic outcome [30]. The 3D images were rotated so that they could be reviewed from either side, cranially looking downwards towards the cleavage and caudally upwards from the feet towards the inferior mammary fold (IMF). The panel scored independently and then the opinion of the majority was assigned as a consensus score. If there was disparity in scoring the images, a discussion ensued to reach a consensus; the method used in other studies with a cosmetic outcome endpoint [31–33]. To evaluate the test–retest variation, 10% of all the images were randomly shown twice and the two scores compared.

**Table 1:** 4-Point Harvard cosmesis scale

Outcome	Description	Score
Excellent	Treated breast nearly identical to untreated breast.	4
Good	Treated breast slightly different from untreated breast.	3
Fair	Treated breast clearly different from untreated breast but not seriously distorted.	2
Poor	Treated breast seriously distorted.	1

### Patient satisfaction

The BREAST-Q is a questionnaire devised by the Memorial Sloan-Kettering Cancer Center to elicit and quantify patient perception of outcomes after breast surgery [12, 34]. It has been developed using extensive patient input and Rasch psychometric methods [35, 36] to measure patient satisfaction and quality of life. Modules have been developed for patients undergoing mastectomy, breast reconstruction and most recently Breast Conserving Therapy (BCT). This module contains eight domains. However, only the ‘Satisfaction with breasts’ domain and some of its sub-questions were used in this study. Each domain contains sub-questions and the ‘raw’ score is transformed to a score ranging from 0 to 100, where a higher score indicates greater satisfaction. We have previously reported results of all of the domains of the BREAST-Q BCT module [37].

### Statistical analysis

Demographics were presented as descriptive statistics using mean and standard deviation or median and IQR range, as appropriate, after testing for normality. Categorical data were presented as proportions and frequencies with 95% confidence intervals (CIs) where appropriate.

The Kruskal–Wallis test was used to assess the relationship between categorical (e.g. panel assessment, sub-questions from ‘Satisfaction with breasts’) and continuous variables (e.g. volume symmetry, shape symmetry, ‘Satisfaction with breasts’), after testing for normality. The Dunn–Sidak test (a post hoc adjusted pairwise comparison) was used to identify between which pairs of categorical results the significant differences lay. Any pairwise comparisons with a *P* < 0.05 were considered statistically significant.

Spearman’s rho correlations were used to assess the relationship between two continuous variables after testing for normality. A correlation coefficient result between 0.00 and 0.019 indicates very weak correlation, 0.20 and 0.39 is weak, 0.40 and 0.59 is moderate, 0.60 and 0.79 is strong and 0.80 and 1.0 is very strong.

A kappa statistic was used for comparison of two categorical datasets. A value of 0 indicates no agreement, 0–0.20 is slight, 0.21–0.40 is fair, 0.41–0.60 is moderate, 0.61–0.80 is substantial, and 0.81–1 is almost perfect. Analysis was undertaken using STATA (STATA, Inc., Texas).

## Results

Between 01/04/2015 and 31/10/2015, 649 women were scheduled to have a surveillance mammogram. Three hundred and forty two (53.7%) women were eligible and had a mammogram booked at a time when the investigator (ROC) was available. All were invited but 109 were not contactable by phone to confirm participation. Of the 233 women who were contactable, 206 (88.4%) agreed to participate and 27 (11.6%) declined. In total, 200 (85.8%) women participated. The clinicopathological characteristics of the study population are summarised in Table 2.

**Table 2:** Summary of study participants’ clinicopathological characteristics

Clinicopathological data	Study population
**Pre-operative data**	
Age at time of surgery (years), mean (SD)	64.2 (10.1)
Time from surgery to study participation (months), mean (SD)	35.6 (17.7)
Ethnic origin (%)	
White	186 (93)
Non-white	14 (7)
Smoking status (%)	
Never	119 (59.5)
Current	16 (8)
Ex-smoker	65 (32.5)
BMI at surgery (kg/m^2^), mean (SD)	27.5 (5.4)
Location of tumour on pre-operative imaging (%)	
Upper outer	109 (54.5)
Central	8 (4)
Lower inner	27 (13.5)
Lower outer	21 (10.5)
Upper inner	35 (17.5)
Ultrasound size (mm), mean (SD)	13.9 (8.6)
Mammographic size (mm), mean (SD)	16.26 (10.88)
**Intra-operative data**	
Type of surgery (%)	
WLE	181 (90.5)
Other complex breast conservation	19 (9.5)
Axillary surgery (%)	
Nil	19 (9.5)
SLNB or sampling	150 (75)
ALND	31 (15.5)
Re-excision of margins (%)	
No	169 (84.5)
Yes	31 (15.5)
Pathology data	
Tumour pathology size including DCIS (mm), mean (SD)	21.6 (13.1)
Weight of specimen (g), median (IQR)	32.5 (20–49)
**Adjuvant therapy**	
Adjuvant chemotherapy (%)	
No	161 (80.5)
Yes	39 (19.5)
Endocrine Therapy (%)	
No	30 (15)
Yes	170 (85)
Whole breast radiotherapy (%)	
No	0
Yes	200 (100)
Boost radiotherapy (%)	
No	149 (74.5)
Yes	51 (25.5)

Median panel score was 3 (IQR 2–4). Eight (4%) participants were assigned a panel score of poor, 62 (31%) scored fair, 78 (39%) scored good and 52 (26%) scored excellent. For the 10% test–retest validation of the panel assessment, the weighted agreement was 98.3%, Kappa 0.96 (almost perfect), (*P* < 0.001). Median score for ‘Satisfaction with breasts’ was 69.5 (IQR 31–100).

**Fig. 2:**
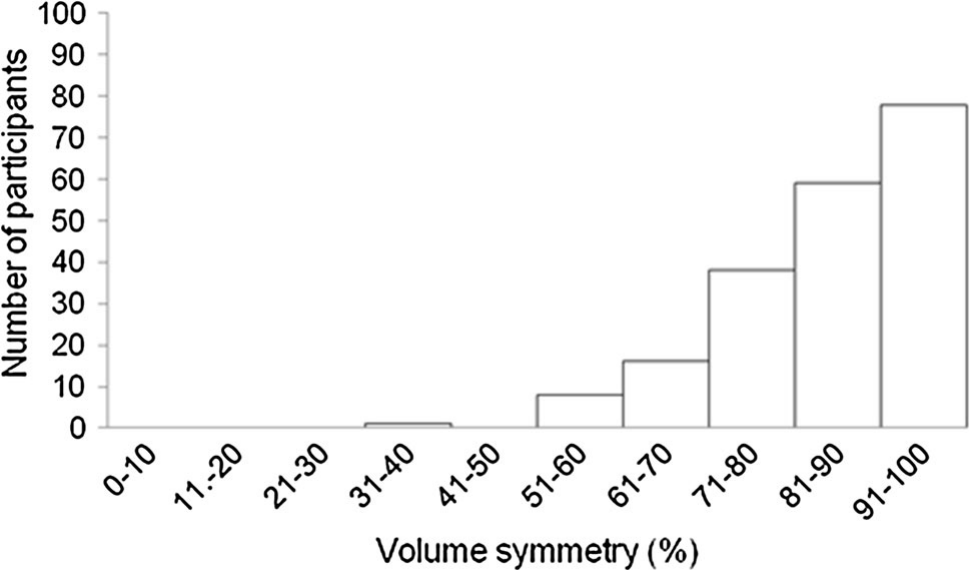
Frequency distribution of volume symmetry

**Fig. 3:**
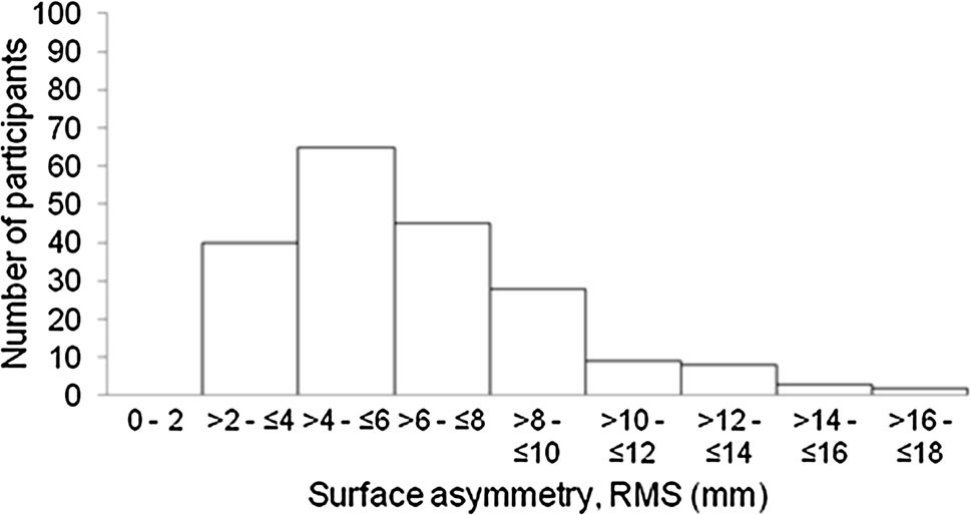
Frequency distribution of shape symmetry, root mean squared (RMS)

The median treated breast volume was 456 cm^3^ (IQR 323–680 cm^3^), median untreated breast volume was 493 cm^3^ (IQR 340–740 cm^3^). The median volume symmetry was 87% (IQR 78–93%) (Fig. 2). The median shape symmetry, RMS, was 5.9 mm (IQR 4.2–8.0 mm) (Fig. 3).

### Relationship of objective 3D-SI measurements of volume and shape symmetry with *panel assessment*

#### Volume symmetry

**Fig. 4:**
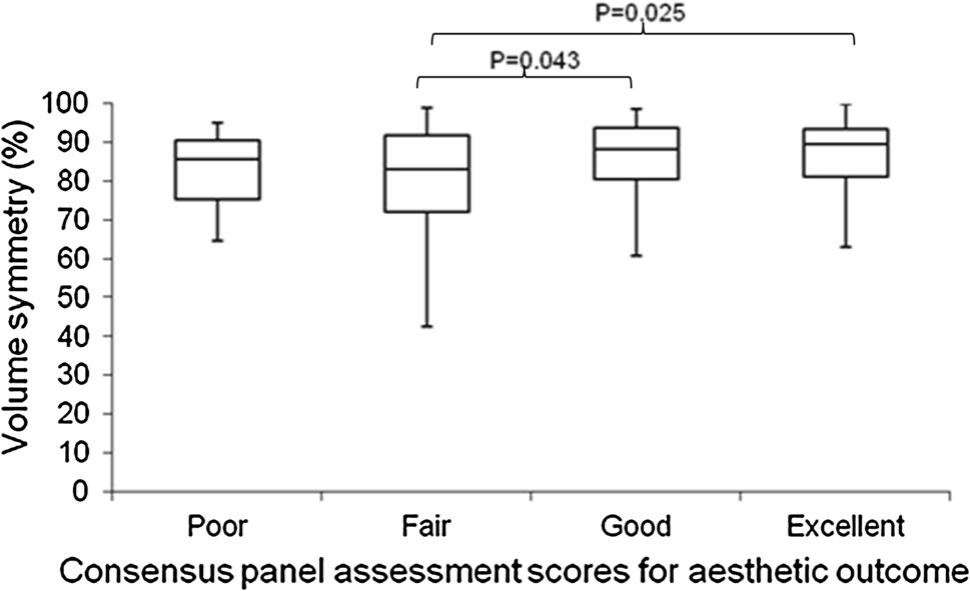
*Box* and *whisker* plot demonstrating volume symmetry (%) according to consensus panel assessment of aesthetic outcome. The *horizontal lines* within each *box* represent median scores, the *outer horizontal lines* of each *box* represent *upper* and *lower* quartiles, and the ends of the *vertical lines* represent minimum and maximum scores. On post hoc pair wise comparisons there was a significant difference in volume symmetry when comparing ‘*fair*’ with ‘*good*’ and ‘*fair*’ with ‘*excellent*’ panel scores. The other comparisons were not significant

The median volume symmetry measurements differed between panel assessment groups (Kruskal–Wallis test, *P* = 0.028) (Table 3). Post hoc pairwise comparison using Dunn–Sidak test further demonstrated that these differences existed between panel scores ‘fair’ and ‘good’, as well as between ‘fair’ and ‘excellent’ (Fig. 4). Participants deemed ‘poor’ by the panel did not have a significantly different volume symmetry from those deemed ‘good’ (*P* = 0.645) or ‘excellent’ (*P* = 0.528). This indicates that a ‘poor’ panel assessment did not necessarily relate to volume symmetry but may be biased by the very small numbers of patients in this category.

**Table 3:** Volume and shape symmetry according to 3D-SI panel scores

Panel assessment consensus scores	Number	Volume symmetry (%) median (IQR)	Shape symmetry (RMS) median (IQR)
1 = Poor	8	85.6 (75.3–90.7)	9.7 (6.5–13.2)
2 = Fair	62	83.1 (72.2–92)	7.9 (6.4–9.8)
3 = Good	78	88.2 (80.6–93.8)	5.2 (4–7.2)
4 = Excellent	52	89.7 (81.3–93.6)	4.6 (3.4–6)
Total	200	87 (78.1–93.4)	5.9 (4.2–8)

#### Shape symmetry

**Fig. 5:**
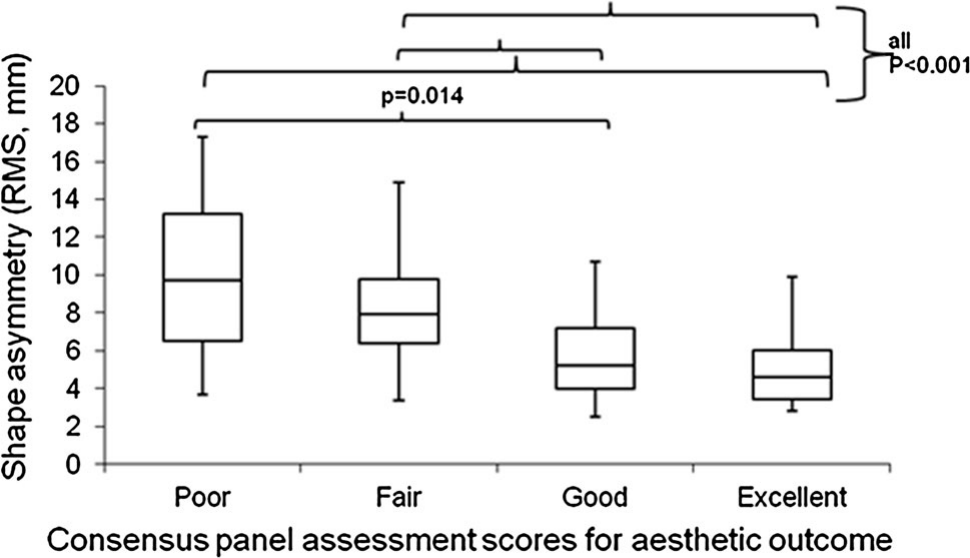
*Box* and *whisker* plot demonstrating shape symmetry RMS, mm) according to consensus panel assessment of aesthetic outcome. The *horizontal lines* within each *box* represent median scores, the *outer horizontal lines* of each *box* represent *upper* and *lower* quartiles, and the ends of the *vertical lines* represent minimum and maximum scores. On post hoc pair wise comparison there was a significant difference in shape symmetry when comparing poor with good, poor with excellent, fair with good and fair with excellent. The other comparisons were not significant

The median shape symmetry measurements differed between panel assessment groups (Kruskal–Wallis test, *P* < 0.001) (Table 3). Post hoc pairwise comparison using the Dunn–Sidak test further demonstrated that these differences arose between panel scores ‘poor’ and ‘good’, ‘poor’ and ‘excellent’, ‘fair’ and ‘good’ as well as ‘fair’ and ‘excellent’ (Fig. 5). This indicates that shape symmetry was always significantly different between those assigned a poor or fair score and those considered to be good or excellent.

### Relationship of objective 3D-SI measurements of volume and shape symmetry with *patient satisfaction*

#### Volume symmetry

There was a significant positive correlation between volume symmetry measurements and ‘Satisfaction with breasts’ scores; however, the correlation was very weak (Spearman’s correlation coefficient = 0.187, *P* = 0.008, *r* ^2^ = 0.033).

#### Shape symmetry

There was a significant negative correlation between shape symmetry measurements and ‘Satisfaction with breasts’ scores; however, the correlation coefficient was also weak (Spearman’s correlation coefficient = −0.229, *P* = 0.001, *r* ^2^ = 0.079).

### Objective volume symmetry and ‘Satisfaction with breasts’ sub-question ‘How equal in size your breasts are to each other?’

Due to the nature of ‘Satisfaction with breasts’ being a global outcome score, the 3D volume and shape symmetry were tested against participants’ perceptions using the sub-questions relating to volume and symmetry.

The median volume symmetry measurements differed between groups according to patient-reported score for ‘How equal in size your breasts are to each other’ (Kruska-Wallis test, *P* < 0.001). Post hoc pairwise comparison using Dunn–Sidak test further demonstrated that there were significant differences between participants’ scores for equality of size ‘very dissatisfied’ and ‘very satisfied’ (*P* = 0.046), ‘somewhat dissatisfied’ and ‘somewhat satisfied’ (*P* = 0.008), ‘somewhat dissatisfied’ and ‘very satisfied’ (*P* = 0.006) (Table 4). This indicates that participants who were ‘very satisfied’ with how equal in size their breasts are had significantly better volume symmetry than those who answered ‘very dissatisfied’ or ‘somewhat dissatisfied’.

### Objective surface symmetry and ‘Satisfaction with breasts’ sub-question ‘How much your breasts look the same?’

The median shape symmetry measurements were significantly different according to patient-reported score for ‘How much your breasts look the same’ (Kruskal–Wallis test, *P* < 0.001). Post hoc pairwise comparison using Dunn–Sidak test further demonstrated that these differences arose between participants’ scores ‘very dissatisfied’ and ‘somewhat satisfied’ (*P* = 0.017), ‘very dissatisfied’ and ‘very satisfied’ (*P* < 0.001), ‘somewhat dissatisfied’ and ‘very satisfied’ (*P* = 0.002) and ‘somewhat satisfied’ and ‘very satisfied’ (*P* = 0.002) (Table 4). This indicates that participants who were ‘very satisfied’ that their breasts look the same had a greater objective symmetry compared to participants who rated it as ‘very dissatisfied’, ‘somewhat dissatisfied’ and ‘somewhat satisfied’.

**Table 4:** Volume symmetry according to ‘How equal in size your breasts are to each other?’ and shape symmetry according to ‘How much your breasts look the same?’

Sub-question Likert scale	With your breasts in mind, in the past 2 weeks, how satisfied or dissatisfied have you been with:			
	How equal in size your breasts are to each other?		How much your breasts look the same?	
	Number	Median volume symmetry (%) median (IQR)	Number	Median shape symmetry (RMS) median (IQR)
1 = Very dissatisfied	11	78.1 (71.1–82.3)	13	7.8 (7.2–10.4)
2 = Somewhat dissatisfied	41	79.9 (69.8–89.4)	40	6.4 (4.6–9.1)
3 = Somewhat satisfied	87	89 (81.7–93.8)	93	6.1 (4.6–8.1)
4 = Very satisfied	61	89.8 (80.9–93.6)	54	4.6 (3.4–6.3)
Total	200	87 (78.1–93.4)	200	5.9 (4.2–8)

## Discussion

This study shows that 3D measures of volume and shape symmetry agree with panel assessment and to a lesser extent with PROMs, indicating that 3D surface imaging could contribute to an objective measure of aesthetic outcome. Currently, the mainstays of analysis of aesthetic outcome are panel assessment of 2D photographs and objective scoring software using 2D photographs. Panel assessments have limitations in the time and manpower required. Furthermore, they are difficult to standardise and there is no assurance that, even if the same scale is used, two panels would score with identical strictness or leniency. Objective analysis offers a potential solution but 2D photographs may not provide the ‘whole story’ due to the very nature of analysing a three-dimensional object in two dimensions. For example, 2D photographs do not demonstrate the projection and cleavage well, whereas in 3D-SI, the image of the patient can be rotated to view the cleavage and allow the reviewer to see the patient as she sees herself.

Patients also provide insight into their own assessment of the aesthetic outcome with PROMs, and this is a key outcome measure. However, there may be many confounding factors in the patients’ responses due to their pre-morbid state and the psychological impact of diagnosis and treatment of breast cancer such that PROMs alone cannot be used in evaluation of new surgical or radiotherapy techniques. We have shown that 3D-SI can contribute to these evaluations.

In this study, there were only two significant pairwise comparisons of volume symmetry out of a possible seven when assessing its relationship with panel assessment. It is possible that surgery and radiotherapy lift the breast on the chest wall so that the panel assessment of symmetry is poor but the objective volume symmetry is relatively good. Similarly, a patient may have a focal deformity, drawing the reviewer’s attention resulting in a low panel score but the overall objective volume is similar to the unaffected breast. Finally, the low association found between panel and volume symmetry may also have been due to the panel method used as the Harvard classification focuses on symmetry and deformity of the breasts rather than volume.

There was a better association between objective shape symmetry and panel score with four significant pairwise comparisons. Shape symmetry encompasses volume as part of the whole, just as a panel will evaluate appearance more globally and the Harvard score focuses on symmetry so it is unsurprising that there was a better association with shape than with volume symmetry. Our findings are in keeping with those of Henseler et al. [38] who found a significant relationship between the mean subjective panel assessment score and objective symmetry (correlation coefficient −0.62) when assessing forty-four patients who had undergone unilateral extended latissimus dorsi flap reconstruction.

Volume symmetry and shape symmetry were both correlated with patient ‘Satisfaction with breasts’. However, these correlations were weak such that these objective measurements are unlikely to replace a patient’s perception of aesthetic outcome. The patient satisfaction domain of the BREAST-Q encompasses many aspects of how the patient feels about the aesthetic outcome and, as mentioned previously, many biases can confound a patients’ satisfaction other than pure aesthetic outcome. These results may also reflect the way in which the BREAST-Q module was developed and it could be that volume and symmetry had low weighting in the overall scoring compared to other factors, for example, how the patient feels about her appearance unclothed. To assess this further, we investigated the relationship between the sub-question ‘How equal in size your breasts are to each other’ and volume symmetry. There were three significant pairwise comparisons indicating that patients’ subjective assessment on a 4-point scale correlates well with objective assessment of volume symmetry. Sub-question ‘How much your breasts look the same’ demonstrated even better association with shape symmetry, with four pairwise comparisons. A similar study was undertaken by Yip et al. [39] where 119 women who had undergone immediate or delayed breast reconstruction underwent 3D-SI and answered the BREAST-Q post-operative reconstruction module. Unlike this study, they found no correlation between volume symmetry and ‘Satisfaction with breasts’ but they did find that patients were able to perceive volume difference on answering the sub-questions. The authors did not assess shape symmetry. The heterogeneity of their patient population may account for the difference in results between their study and ours.

We believe that 3D-SI will have an important role in the evaluation of breast surgery and radiotherapy in the future. As the technology evolves to become more portable and lower cost, [40–44] it will be available to more units treating patients with breast cancer, and could be used as a robust outcome tool in multicentre surgical and radiotherapy studies as well as in internal audits and quality assurance studies.

## Conclusion

Breast volume and shape symmetry measured using 3D-SI are both associated with panel assessment of breast appearance and patient satisfaction with the cosmetic outcome. Shape symmetry, in particular, showed greatest association with panel assessment, such that it may be possible to replace this with an objective outcome score encompassing shape symmetry and other parameters measured using 3D-SI.

## References

[1] Lawrence GKO, Lagord C, Cheung S, Sidhu J, Sagar C (2011) The second all breast cancer report. Focussing on inequalities: variation in breast cancer outcomes with age and deprivation. National Cancer Intelligence Network, London

[2] Fisher B, Jeong JH, Anderson S, Bryant J, Fisher ER, Wolmark N (2002). Twenty-five-year follow-up of a randomized trial comparing radical mastectomy, total mastectomy, and total mastectomy followed by irradiation. N Engl J Med.

[3] Veronesi U, Banfi A, Del Vecchio M, Saccozzi R, Clemente C, Greco M (1986). Comparison of Halsted mastectomy with quadrantectomy, axillary dissection, and radiotherapy in early breast cancer: long-term results. Eur J Cancer Clin Oncol..

[4] UK CR (2013) Breast Cancer Statistics: Cancer Research UK

[5] Al-Ghazal SK, Fallowfield L, Blamey RW (1999). Does cosmetic outcome from treatment of primary breast cancer influence psychosocial morbidity?. Eur J Surg Oncol.

[6] Waljee JF, Hu ES, Ubel PA, Smith DM, Newman LA, Alderman AK (2008). Effect of esthetic outcome after breast-conserving surgery on psychosocial functioning and quality of life. J Clin Oncol.

[7] Heil J, Holl S, Golatta M, Rauch G, Rom J, Marmé F (2010). Aesthetic and functional results after breast conserving surgery as correlates of quality of life measured by a German version of the Breast Cancer Treatment Outcome Scale (BCTOS). Breast.

[8] Cardoso MJ, Oliveira H, Cardoso J (2014). Assessing cosmetic results after breast conserving surgery. JSurgOncol.

[9] Chen CM, Cano SJ, Klassen AF, King T, McCarthy C, Cordeiro PG (2010). Measuring quality of life in oncologic breast surgery: a systematic review of patient-reported outcome measures. Breast J..

[10] Cohen WA, Mundy LR, Ballard TN, Klassen A, Cano SJ, Browne J (2015). The BREAST-Q in surgical research: a review of the literature 2009–2015. J Plast Reconstr Aesthet Surg.

[11] Kanatas A, Velikova G, Roe B, Horgan K, Ghazali N, Shaw RJ (2012). Patient-reported outcomes in breast oncology: a review of validated outcome instruments. Tumori.

[12] Pusic AL, Chen CM, Cano S, Klassen A, McCarthy C, Collins ED (2007). Measuring quality of life in cosmetic and reconstructive breast surgery: a systematic review of patient-reported outcomes instruments. Plast Reconstr Surg.

[13] Harris JR, Levene MB, Svensson G, Hellman S (1979). Analysis of cosmetic results following primary radiation therapy for stages I and II carcinoma of the breast. Int J Radiat Oncol Biol Phys.

[14] Sneeuw KC, Aaronson NK, Yarnold JR, Broderick M, Regan J, Ross G (1992). Cosmetic and functional outcomes of breast conserving treatment for early stage breast cancer. 1. comparison of patients’ ratings, observers’ ratings, and objective assessments. Radiother Oncol.

[15] Fitzal F, Krois W, Trischler H, Wutzel L, Riedl O, Kuhbelbock U (2007). The use of a breast symmetry index for objective evaluation of breast cosmesis. Breast.

[16] Cardoso MJ, Cardoso J, Amaral N, Azevedo I, Barreau L, Bernardo M (2007). Turning subjective into objective: the BCCT.core software for evaluation of cosmetic results in breast cancer conservative treatment. Breast.

[17] Cardoso JS, Cardoso MJ (2007). Towards an intelligent medical system for the aesthetic evaluation of breast cancer conservative treatment. Artif Intell Med.

[18] Hidalgo DA, Sinno S (2016). Current trends and controversies in breast augmentation. Plast Reconstr Surg.

[19] O’Connell RL, Stevens RJ, Harris PA, Rusby JE (2015). Review of three-dimensional (3D) surface imaging for oncoplastic, reconstructive and aesthetic breast surgery. Breast.

[20] Kovacs L, Eder M, Hollweck R, Zimmermann A, Settles M, Schneider A (2007). Comparison between breast volume measurement using 3D surface imaging and classical techniques. Breast.

[21] Henseler H, Khambay BS, Bowman A, Smith J, Paul SJ, Oehler S (2011). Investigation into accuracy and reproducibility of a 3D breast imaging system using multiple stereo cameras. J Plast Reconstr Aesthet Surg.

[22] Yip JM, Mouratova N, Jeffery RM, Veitch DE, Woodman RJ, Dean NR (2012). Accurate assessment of breast volume: a study comparing the volumetric gold standard (direct water displacement measurement of mastectomy specimen) with a 3D laser scanning technique. Ann Plast Surg.

[23] Losken A, Seify H, Denson DD, Paredes AA, Carlson GW (2005). Validating three-dimensional imaging of the breast. Ann Plast Surg.

[24] Rao AK, Karanas Y, Galdino GM, Girod SC (2003) Three-dimensional photography for calculating pre- and post-operative volume in breast surgery. In: Lemke HU, Inamura K, Doi K, Vannier MW, Farman AG, Reiber JHC (eds) Cars 2003: computer assisted radiology and surgery, proceedings. International congress series, vol, 1256, p 1355

[25] Koch MC, Adamietz B, Jud SM, Fasching PA, Haeberle L, Karbacher S (2011). Breast volumetry using a three-dimensional surface assessment technique. Aesthet Plast Surg.

[26] Veitch D, Burford K, Dench P, Dean N, Griffin P (2012). Measurement of breast volume using body scan technology(computer-aided anthropometry). Work.

[27] Thomson JG, Liu YJ, Restifo RJ, Rinker BD, Reis A (2009). Surface area measurement of the female breast: phase I. Validation of a novel optical technique. Plast Reconstr Surg.

[28] Lewis P, Mattison G, Kim H, Gupta S (2014). Evaluation of 3D photographic imaging to measure differential volumes in reconstructed breast tissue. J Invest Med.

[29] Henseler H, Smith J, Bowman A, Khambay BS, Ju X, Ayoub A (2012). Investigation into variation and errors of a three-dimensional breast imaging system using multiple stereo cameras. J Plast Reconstr Aesthet Surg.

[30] Rose MA, Olivotto I, Cady B, Koufman C, Osteen R, Silver B (1989). Conservative surgery and radiation therapy for early breast cancer. Long-term cosmetic results. Arch Surg.

[31] Haviland JS, Owen JR, Dewar JA, Agrawal RK, Barrett J, Barrett-Lee PJ (2013). The UK Standardisation of Breast Radiotherapy (START) trials of radiotherapy hypofractionation for treatment of early breast cancer: 10-year follow-up results of two randomised controlled trials. Lancet Oncol.

[32] Bentzen SM, Agrawal RK, Aird EG, Barrett JM, Barrett-Lee PJ, Bliss JM (2008). The UK Standardisation of Breast Radiotherapy (START) Trial B of radiotherapy hypofractionation for treatment of early breast cancer: a randomised trial. Lancet.

[33] Bentzen SM, Agrawal RK, Aird EG, Barrett JM, Barrett-Lee PJ, Bliss JM (2008). The UK Standardisation of Breast Radiotherapy (START) Trial A of radiotherapy hypofractionation for treatment of early breast cancer: a randomised trial. Lancet Oncol.

[34] Pusic AL, Klassen AF, Scott AM, Klok JA, Cordeiro PG, Cano SJ (2009). Development of a new patient-reported outcome measure for breast surgery: the BREAST-Q. Plast Reconstr Surg.

[35] Aaronson N, Alonso J, Burnam A, Lohr KN, Patrick DL, Perrin E (2002). Assessing health status and quality-of-life instruments: attributes and review criteria. Qual Life Res.

[36] US Food and Drug Administration. Guidance for industry. Patient-reported outcome measures: use in medical product development to support labelling claims. Silver Spring (MD). 2009. http://www.fda.gov/downloads/Drugs/./Guidances/UCM193282.pdf. Accessed 3 Jan 2016

[37] O’Connell RL, Di Micco R, Khabra K, O’Flynn EA, deSouza N, Roche N (2016). Initial experience of the BREAST-Q breast-conserving therapy module. Breast Cancer Res Treat.

[38] Henseler H, Smith J, Bowman A, Khambay BS, Ju X, Ayoub A (2013). Subjective versus objective assessment of breast reconstruction. J Plast Reconstr Aesthet Surg.

[39] Yip JM, Watson DI, Tiggemann M, Hsia S, Smallman AE, Dean NR (2015). Determinants of breast reconstruction outcome: how important is volume symmetry?. J Plast Reconstr Aesthet Surg.

[40] Henseler H, Kuznetsova A, Vogt P, Rosenhahn B (2014). Validation of the Kinect device as a new portable imaging system for three-dimensional breast assessment. J Plast Reconstr Aesthet Surg.

[41] Hoeffelin H, Jacquemin D, Defaweux V, Nizet JL (2014). A methodological evaluation of volumetric measurement techniques including three-dimensional imaging in breast surgery. Biomed Res Int.

[42] Patete P, Riboldi M, Spadea MF, Catanuto G, Spano A, Nava M (2009). Motion compensation in hand-held laser scanning for surface modeling in plastic and reconstructive surgery. Ann Biomed Eng.

[43] Wheat JS, Choppin S, Goyal A (2014). Development and assessment of a Microsoft Kinect based system for imaging the breast in three dimensions. Med Eng Phys.

[44] Pöhlmann ST, Hewes J, Williamson AI, Sergeant JC, Hufton A, Gandhi A, Taylor CJ, Astley SM (2014). Breast volume measurement using a games console input device, breast imaging. breast volume measurement using a games console input device. Breast Imaging.

